# Histone variants at a glance

**DOI:** 10.1242/jcs.244749

**Published:** 2021-03-26

**Authors:** Paul B. Talbert, Steven Henikoff

**Affiliations:** Howard Hughes Medical Institute, Fred Hutchinson Cancer Research Center, 1100 Fairview Avenue N, Seattle, WA 98109, USA

**Keywords:** Chromatin, Epigenetics, Replacement histones

## Abstract

Eukaryotic nucleosomes organize chromatin by wrapping 147 bp of DNA around a histone core particle comprising two molecules each of histone H2A, H2B, H3 and H4. The DNA entering and exiting the particle may be bound by the linker histone H1. Whereas deposition of bulk histones is confined to S-phase, paralogs of the common histones, known as histone variants, are available to carry out functions throughout the cell cycle and accumulate in post-mitotic cells. Histone variants confer different structural properties on nucleosomes by wrapping more or less DNA or by altering nucleosome stability. They carry out specialized functions in DNA repair, chromosome segregation and regulation of transcription initiation, or perform tissue-specific roles. In this Cell Science at a Glance article and the accompanying poster, we briefly examine new insights into histone origins and discuss variants from each of the histone families, focusing on how structural differences may alter their functions.

## Abstract (precis)

**Summary:** Histone variants change the structural properties of nucleosomes by wrapping more or less DNA, altering nucleosome stability or carrying out specialized functions.

## Introduction

Eukaryotic chromatin is organized by nucleosomes, which package and regulate access to DNA and whose primary role may be to globally repress transcription (Kornberg and Lorch, 2020). Nucleosomes are formed from an octameric core particle of two molecules each of the histones H2A, H2B, H3 and H4, which wraps 147 bp of DNA (Luger et al., 1997) (see poster). Histone fold domain proteins (HFDs) have a long history in all domains of life (Box 1), but a eukaryotic innovation is their ability to heterodimerize in specific pairs, H3 with H4, and H2A with H2B, which can further associate through four-helix bundles, H3 with H3, and H4 with H2B, to form a central H3–H4 tetramer flanked by two H2A–H2B dimers (see poster). The HFDs have unstructured tails that are the sites of post-translational modifications. A nucleosome may be further stabilized by the H1 ‘linker’ histone, which interacts with the DNA that links adjacent nucleosomes. ‘Bulk’ or ‘replication-coupled’ (RC) histones are primarily deposited during replication. In animals, RC histones are encoded by multiple genes with special mRNAs that lack introns and polyadenylated (polyA) tails, but instead have a 3′ stem-loop structure (Marzluff, 2005). A subset of these genes can also form transcripts with polyA tails in differentiated tissues that no longer replicate, and therefore are available to replace evicted histones (Lyons et al., 2016). Some histone genes, however, encode distinct paralogs that differ in amino acid sequence from their RC counterparts. These histone ‘variants’ are usually encoded by single genes that have both introns and polyA tails, and are typically available for deposition throughout the cell cycle [replication-independent (RI)], replacing evicted RC histones. Histone variants often confer different structural properties on nucleosomes and often have distinct functions in cell division, transcription, DNA repair, differentiation and chromatin remodeling. We have previously reviewed the phylogenomic scope and dynamics of histone variants (Talbert and Henikoff, 2010, 2014, 2017). In this Cell Science at a Glance article, and the accompanying poster, we instead focus on recent insights into histone origins, and present an overview of variants in each histone class, emphasizing mammalian variants.

**Figure:**
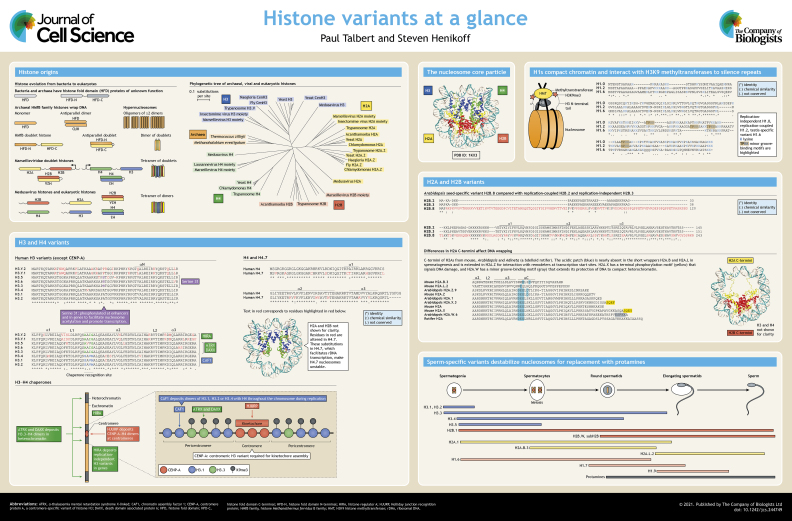

Glossary **+1 nucleosome:** the first nucleosome of a gene, immediately downstream of the nucleosome-depleted region at the transcription start site.

**Azoospermia:** absence of motile sperm in the semen. It can be due to an obstruction of the reproductive tract or to the failure to produce sperm.

**Blastopore:** a mouth-like indentation or opening into the developing gastrula. In vertebrates, it becomes the anus.

**Cohesins:** ring-like protein complexes that hold loops of DNA or sister chromatids together.

**HMfB family:** a family of HFD proteins in archaea (named for histone *Methanothermus fervidus* B) that can wrap DNA but have no significant primary sequence similarity with to eukaryotic histones. They can dimerize, form tetramers or form more extended oligomers.

**Protamines:** small arginine-rich proteins that replace histones in sperm of many vertebrates to further condense the chromatin, presumably to improve sperm motility and DNA protection. Similar proteins are found in many invertebrates.

**Rapid response genes:** genes typically activated within minutes of some stimulus.

**Sex body:** the condensed X and Y chromosomes in mammalian spermatocytes, as the visual manifestation of undergoing meiotic sex chromosome inactivation.

Box 1. Histone origins Histone fold domain (HFD) proteins are found in all three cellular domains and in some viruses (see poster), although two families of HFD proteins that are found in both bacteria and archaea have unknown functions and DNA-binding abilities (Alva and Lupas, 2019). Many archaea additionally have one or more histones of the histone *Methanothermus fervidus* B (HMfB) family, which bind to DNA and are more similar in structure to eukaryotic histones, although they are not specifically related in sequence and mostly lack the unstructured tails of eukaryotic histones (Henneman et al., 2018). HMfB family histones fold together, usually in homomeric dimers, which can be further polymerized through four-helix bundles to form tetrameric HFD particles that wrap 60 bp of DNA or to form more extended DNA-wrapping polymers (Mattiroli et al., 2017), termed hypernucleosomes (Henneman et al., 2018) (see poster). Some also have tandemly coupled, diverged HFDs that fold together and then dimerize into particles with four HFDs. A similar organization of pairs of tandem HFDs has recently been found in Marseilleviridae, where the tandem HFDs have tails and have distant similarity with H4–H3 and H2B–H2A (Erives, 2017). Medusavirus (Yoshikawa et al., 2019), which belongs to the same large clade of nucleo-cytoplasmic large DNA viruses as the Marseilleviridae, encodes individual core histones specifically related to H2A, H2B, H3 and H4. The individual viral histone domains branch in a phylogenetic tree at the base of all eukaryotic histones of the corresponding histone family (see poster), suggesting these viral histones represent an intermediate state in the evolution of histones from archaeal-like HFDs to modern eukaryotic histones. It seems likely that these viruses captured histones at different times in the evolution of proto-eukaryotic histones and adapted them to package their own genomes. Medusavirus also has an H1 linker histone, but it is most closely related (43% identical) to the H1 of its protist host *Acanthamoeba castellani*, suggesting a more recent derivation than the core histones, which do not show a specific relationship to *Acanthamoeba*.

## H3 variants

Most multicellular and some unicellular eukaryotes have separate RC and RI H3 paralogs (Postberg et al., 2010; Waterborg, 2012), although in organisms with only one form of H3, such as yeast, that form is deposited both during and outside of replication and more closely resembles RI variants in sequence (Ahmad and Henikoff, 2002a). In most animals, the RC and RI H3s correspond to human H3.2 and H3.3, respectively. Human H3.1, which differs in a single amino acid from H3.2, is a mammal-specific RC paralog (Postberg et al., 2010). Differences between RC and RI H3s in residues 87–90 in the N-terminal end of α-helix 2 (SAVM versus AAIG in animals) specify different modes of chromatin assembly (Ahmad and Henikoff, 2002a,b) mediated by different chaperones: the chromatin assembly factor 1 (CAF1; CHAF1A, CHAF1B and RBBP4) complex for RC H3.1–H4 dimers, and histone regulator protein A (HIRA) (Tagami et al., 2004), or α-thalassemia mental retardation syndrome X-linked (ATRX) and death domain-associated protein 6 (DAXX) (Elsässer et al., 2012) for RI H3.3–H4 dimers (see poster). Whereas CAF1 distributes H3.1 and H3.2 throughout the genome during replication, HIRA replaces nucleosomes lost during transcription with H3.3 nucleosomes, thereby depositing H3.3 in active genes, promoters, enhancers, transcription termination sites and other locations of histone turnover (Mito et al., 2005, 2007; Wirbelauer et al., 2005). ATRX and DAXX deposit H3.3 in telomeres, imprinted genes and other heterochromatic loci, where it is modified with the trimethlyated lysine 9 heterochromatic mark (denoted H3K9me3) to maintain heterochromatin at these locations (Drane et al., 2010; Elsässer et al., 2015; Goldberg et al., 2010; Voon et al., 2015; Wong et al., 2010). The difference between deposition pathways for RC H3.1 and RI H3.3 underlies the more-severe effects of pediatric diffuse midline gliomas caused by H3.1K27M mutations than those caused by H3.3K27M mutations, both of which inhibit global formation of H3K7me3, which normally prevents tumorigenesis (Sarthy et al., 2020).

Although *Caenorhabditis* H3.3 is not required for viability (Delaney et al., 2018), in H3.3-deficient *Drosophila* males, chromosomes fail to condense properly for meiosis and undergo segregation defects (Sakai et al., 2009). In mice with H3.3 knockout mutations, failure to maintain heterochromatin leads to mitotic abnormalities and embryonic lethality (Jang et al., 2015). In H3.3-reduced male mice, apoptosis of spermatogonia and spermatocytes occurs, and the transition from histones to protamines (see Glossary) during spermatogenesis is incomplete (Yuen et al., 2014).

RI H3.3s also typically differ from RC H3s in having a serine or threonine residue in the N-terminal tail (serine 31 in animals) (Waterborg and Robertson, 1996), which is phosphorylated in the pericentromere (Hake et al., 2005) and at telomeres (Wong et al., 2009) during metaphase by checkpoint kinase 1 (CHK1; also known as CHEK1) and Aurora B kinases (Chang et al., 2015; Li et al., 2017). In euchromatin of mouse embryonic stem cells, phosphorylation of H3.3S31 (H3.3S31ph) by CHK1 promotes p300-dependent acetylation at enhancers, which facilitates differentiation of these cells (Martire et al., 2019). In *Xenopus* embryos, H3.3S31ph is necessary for blastopore (see Glossary) closure, and nucleosomes with the phosphomimic H3.3S31D are enriched for H3.3K27 acetylation, which is permissive for gene activation (Sitbon et al., 2020). In addition, in mouse cells, such as macrophages, stimulated to rapid response (see Glossary), H3.3S31 is co-transcriptionally phosphorylated over stimulation-induced gene bodies and interacts directly with the histone lysine N-methyltransferase protein SETD2 to promote H3K36me3 formation in genes and to eject the co-repressor ZMYND11, enhancing transcription (Armache et al., 2020).

In *Arabidopsis*, H3.3T31 inhibits the H3K27 methyltransferases *Arabidopsis* trithorax-related proteins 5 and 6 (ATXR5 and ATXR6). ATXR5 and ATXR6 themselves recognize H3.1A31 and methylate K27 to assure this heterochromatic mark is inherited through replication while avoiding silencing active chromatin containing H3.3 (Jacob et al., 2014). Similarly, the H3.3-like sperm-specific variant H3.10 is altered near K27 and not recognized by ATXR5 or ATXR6, contributing to the loss of H3K27me3 and the expression of spermatogenesis genes (Borg et al., 2020). This suggests that a serine or threonine at residue 31 of H3.3 may be conserved across eukaryotic kingdoms to facilitate nucleosome acetylation and enhanced access for transcriptional machinery and to prevent silencing.

Human H3.Y.1 and H3.Y.2 (H3.X) are H3.3-like variants expressed in early cleavage-stage embryos, where they are induced by brief expression of the double homeobox protein 4 (DUX4) transcription factor (Resnick et al., 2019). They become incorporated into DUX4-inducible genes and promote perdurance of expression of these genes. Similar to what is found for the H3R42H change in mouse H3.4 (Table 1), the H3R42K change in H3.Y.1 and H3.Y.2 create more flexible DNA ends that bind histone H1 less efficiently in both homotypic H3.Y.1 nucleosomes and heterotypic H3.Y.1-H3.3 nucleosomes (Kujirai et al., 2016). Despite having a chaperone recognition sequence identical to H3.3 in the α2-helix, H3.Y.1 and H3.Y.2 nucleosomes are only deposited by HIRA and not by DAXX, which requires the C-terminus of H3.3 (Zink et al., 2017).

**Table 1.**
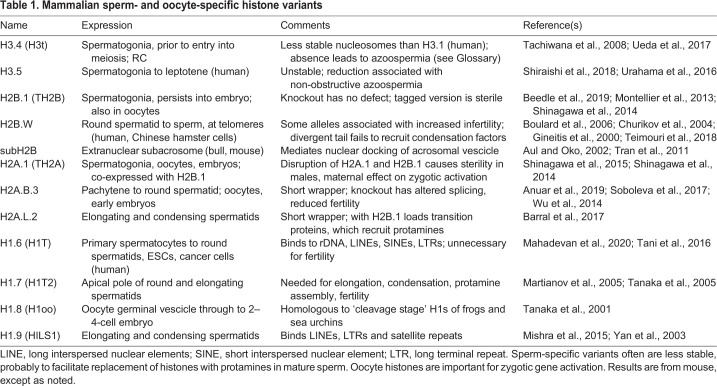
**Mammalian sperm- and oocyte-specific histone variants**

Other human variants, H3.6, H3.7 and H3.8, are tissue-specific (Taguchi et al., 2017). H3.6 forms nucleosomes that seem to have a deposition pattern similar to H3.3 nucleosomes, but are less stable because of an I62V change that reduces hydrophobic contact with H4. H3.7 does not form nucleosomes *in vitro* and H3.8 has a very low but detectable expression level in ovary, colon and kidney (Taguchi et al., 2017). Tissue-specific H3 variants are fairly common in ciliates, plants and animals (Ingouff et al., 2007; Maehara et al., 2015; Moosmann et al., 2011; Postberg et al., 2010), and are often expressed during animal spermatogenesis, where they may destabilize nucleosomes in the transition from histones to protamines (Table 1).

The most divergent and universal H3 variant is the centromere-specific variant cenH3, known as Cse4 in budding yeast, CENP-A in animals and CENH3 in plants. cenH3 serves as a foundational protein required to build the kinetochore in most eukaryotes, but surprisingly it has been lost in trypanosomes (Akiyoshi and Gull, 2014), the fungus *Mucor* (Navarro-Mendoza et al., 2019) and in four clades of holocentric insects (Drinnenberg et al., 2014), which comprise thousands of species. The HFDs of cenH3s are generally only ∼50% conserved with those of other H3s and their N-terminal tails and loop 1 are typically longer than H3 tails and cannot be aligned between divergent species (Talbert et al., 2008). Distinct chaperones exist for cenH3s (Camahort et al., 2007; Chen et al., 2014; Dunleavy et al., 2009; Mizuguchi et al., 2007; Stoler et al., 2007), which, at least in *Drosophila*, recognize loop 1 and co-evolve with it (Rosin and Mellone, 2016). Unlike most other histones, cenH3s in animals and plants evolve rapidly, presumably because competition between centromeres for inclusion in the egg in female meiosis drives rapid evolution of centromeres (Henikoff et al., 2001). This phenomenon, known as centromere drive, can result in the expansion of centromeres (reviewed in Kursel and Malik, 2018; Rosin and Mellone, 2017) and/or in centromeres with a 10 bp periodicity of A/T dinucleotides, which favor stable wrapping of nucleosomes (Talbert and Henikoff, 2020). cenH3s are thought to then evolve to restore equal segregation of unequal centromeres. This conflict highlights the evolutionary tension between genetic (DNA-dependent) and epigenetic (DNA-sequence-independent) pressures on cenH3 evolution (Dawe and Henikoff, 2006).

In summary, from a likely single H3.3-like ancestor, H3 paralogs diversified to specialize for roles in replication, kinetochore formation and spermatogenesis and for differential modification to promote or resist gene silencing. H3 variants can differ in their stability, their modifications, the enzymes or structures that interact with them, or, through their chaperones, differ in where and when they are assembled into nucleosomes.

## H4 variants

H4 variants are infrequent, but humans have H4.7 (H4.G), which has a truncated C-terminus and only 85% amino acid identity with RC H4 (Long et al., 2019). The *H4C7* gene is encoded in the histone 1 gene cluster along with RC histones, but forms a polyadenylated transcript that is expressed at low, but elevated, levels in breast and colon cancer cell lines relative to normal breast tissue (Long et al., 2019). H4.7 localizes to the nucleolus through an interaction of its α3-helix with nucleophosmin 1 and appears to form unstable nucleosome-like structures and less compact chromatin on rDNA, which promotes rRNA transcription (Pang et al., 2020).

## H2B variants

Whereas H3 and H4 RC genes usually encode only one or a few distinct proteins, RC H2A and H2B genes often encode several different proteins. In *Arabidopsis*, ten H2Bs differ mostly in the lysine-rich N-terminal tails and in tissue-specific expression (Bergmuller et al., 2007). Although most appear to be RC H2Bs, H2B.3 is enriched in mature leaves and in nucleosomes containing H3.3 and/or H2A.Z, consistent with it being a RI variant, and H2B.8 is enriched in dry seed (Jiang et al., 2020) (see poster). A similar heterogeneity of RC H2B and H2A proteins is found in humans, mice and sea urchins (Marzluff et al., 2002, 2006). In mice, the mRNA for H2B.21 (H2B.E), which differs in five amino acids from the H2B consensus, is transcribed from the HIST2 gene cluster of RC histones but has a polyA tail, typical of RI variants, and is expressed exclusively in the main olfactory epithelium and the vomeronasal organ (Santoro and Dulac, 2012). A model of H2B.21 function proposes that increased neuronal activity due to exposure to olfactory stimulants reduces H2B.21 in the corresponding olfactory receptor cells and increases neuronal longevity, whereas stimulant deprivation leads to increased H2B.21 levels and shorter neuronal life span. Other H2B variants are mainly restricted to roles in spermatogenesis (Table 1).

## H2A variants

H2A variants occupy the entry and exit positions along the wrap of nucleosomal DNA, making them ideal components to control access to the DNA. Bulk RC H2As in humans differ mostly at their C-termini (Marzluff et al., 2002). The mammalian RI variant H2A.22 (H2A.J) differs from bulk H2As because of an A11V change in the N-terminus and by several residues at its C-terminus, including a potential SQ phosphorylation site, and promotes senescence-associated inflammatory gene expression in cells with persistent DNA damage (Contrepois et al., 2017). Other RI variants also differ at their C-termini, which can alter the extent of DNA wrapping (Doyen et al., 2006b; Osakabe et al., 2018) (see poster). They may also differ at the acidic patch, which interacts with chromatin remodelers, and in loop 1, where the two copies of H2A in a nucleosome contact each other to stabilize nucleosomes (Osakabe et al., 2018).

### H2A.X – DNA damage and beyond

H2A.X differs from bulk H2As in possessing a C-terminal SQD/EΦ phosphorylation motif (where Φ indicates a hydrophobic residue, often phenylalanine or tyrosine). In response to DNA damage, the serine (S139 in humans) becomes phosphorylated (then known as γH2A.X) and recruits repair enzymes to double-strand breaks (reviewed in Talbert and Henikoff, 2014). In single-celled eukaryotes, such as yeast, H2A.X can be the primary form of H2A, whereas in multicellular eukaryotes H2A.X is usually closely related to RC H2As in the same group of multicellular organisms. Because of its conserved function in the DNA damage response, it seems probable that H2A.X is ancestral, and that the various RC H2As are derived from it (Talbert and Henikoff, 2010). In humans, H2A.X differs from other H2A variants in producing mRNAs with either a stem-loop structure typical of RC H2As or a polyadenylated transcript like RI variants (Mannironi et al., 1989). The stem-loop structure may be relevant to deposition of H2A.X during UVC-induced DNA damage repair (Piquet et al., 2018), where it may serve to augment the amount of H2A.X available to be phosphorylated. Sites prone to DNA damage in cycling cells become enriched with H2A.X, whereas resting cells do not show such enrichment (Seo et al., 2012). γH2A.X stimulates the polymerase activity of poly-ADP-ribose polymerase 1 (PARP1), which detects double-strand breaks and recruits additional repair factors (Sharma et al., 2019).

The functions of H2A.X are not limited to DNA repair. In mouse embryonic stem cells, H2A.X is deposited at rDNA promoters and recruits the nucleolar remodeling complex to repress rDNA transcription and limit cell proliferation (Eleuteri et al., 2018), independently of S139 phosphorylation. In *Xenopus*, a variant of H2A.X (H2A.X-F) is abundant and is phosphorylated in oocytes, eggs and early embryos in the absence of DNA damage (Shechter et al., 2009), suggesting it has a role in promoting the rapid early divisions or in activating the zygotic genome. H2A.X-knockout mice are viable but show repair defects and genomic instability, and males are infertile (Celeste et al., 2002), presumably because the X and Y chromosomes in spermatocytes fail to form the sex body (see Glossary) or initiate meiotic sex chromosome inactivation (Fernandez-Capetillo et al., 2003).

### H2A.Z – a transcriptional regulator

The RI variant H2A.Z has a prominent and complex role in transcriptional regulation (reviewed in Giaimo et al., 2019). It is absent from the early-diverging metamonads *Giardia* and *Trichomonas* (Dalmasso et al., 2011), but is strongly conserved in nearly all other eukaryotes, where it commonly occurs in the +1 nucleosome position (see Glossary) of genes (Raisner et al., 2005) and appears to poise genes for transcription and promote RNA polymerase II (RNAPII) recruitment (Adam et al., 2001). H2A.Z has an extended acidic patch that stimulates ATP-dependent remodelers (Dann et al., 2017; Goldman et al., 2010). The yeast SWR1 complex replaces H2A–H2B dimers with H2A.Z–H2B dimers in the +1 nucleosome of transcribed genes (Ranjan et al., 2013) and even in transcribed upstream antisense noncoding RNAs (Bagchi et al., 2020). *In vitro*, H2A.Z nucleosomes have a lower breaking force in an optical tweezer assay (Rudnizky et al., 2016) than bulk H2A nucleosomes. This may underlie the ability of H2A.Z to lower the barrier to transcription of the +1 nucleosome in *Drosophila* cells (Weber et al., 2014), which occurs through the loss of an H2A.Z–H2B dimer and its DNA contacts (Ramachandran et al., 2017), as well as its lower thermal stability (Osakabe et al., 2018). This presumably facilitates the eviction of H2A.Z without loss of H3 in the thermal response of *Arabidopsis* (Cortijo et al., 2017). In yeast, eviction of H2A.Z is dependent on the transcription pre-initiation complex (Tramantano et al., 2016) and serine-5-phophorylated RNAPII (Wu et al., 2009). Apparently contradictory effects of H2A.Z on gene activation or silencing in different contexts are mediated at least in part by acetylation of the N-terminus or monoubiquitylation of the C-terminus, respectively (Giaimo et al., 2019). H2A.Z is also enriched at enhancers, where it is necessary for recruitment of RNAPII and cohesins (see Glossary), which mediate enhancer–promoter interaction, and for the transcription of enhancer RNAs (Brunelle et al., 2015).

Chordates encode two H2A.Z proteins (Eirín-López et al., 2009), H2A.Z.1 and H2A.Z.2, which differ by three conserved amino acids (reviewed in Cheema et al., 2020). H2A.Z.1-knockout animals die during early development (Faast et al., 2001) and H2A.Z.2 is required for melanocyte development in zebrafish (Raja et al., 2020). In humans, H2A.Z.1 and H2A.Z.2 have qualitatively similar, but quantitatively different, expression patterns, with a subset of H2A.Z.2-biased enhancers affecting genes that are downregulated in the cranio-facial abnormality disease floating harbor syndrome (Greenberg et al., 2019). In primates, H2A.Z.2 has two splice variants, H2A.Z.2.1 and H2A.Z.2.2 (Bonisch et al., 2012). H2A.Z.2.2 destabilizes nucleosomes due to its shorter C-terminus, which resembles short H2As in length (Table 1). Despite the requirements for H2A.Z.1 in embryonic development, and the role of H2A.Z.2 in melanocyte development and cranio-facial formation, a double knockout of these two genes in mouse skeletal muscle has little effect on either basal or induced transcription, calling into question whether H2A.Z plays any necessary part in transcription or simply has a replacement function (Belotti et al., 2020). The strong conservation of H2A.Z at transcription start sites across diverse eukaryotic kingdoms is hard to rationalize if it has no role in transcription, since other replacement H2As, such as H2A.X are readily available, in most cases. However, if the role of H2A.Z is to help attract RNAPII or reduce the barrier of the +1 nucleosome, there are likely redundant pathways and cofactors for accomplishing this.

### H2A.W and macroH2A

*Arabidopsis thaliana* has four H2A variants – bulk H2A, H2A.X, H2A.Z and the plant-specific H2A.W – all of which form homotypic nucleosomes (Osakabe et al., 2018). With its extended C-terminus (which has a putative minor groove-binding motif KSPKK), H2A.W protects an additional 10–15 bp of linker DNA beyond the 147 bp of most nucleosomes from micrococcal nuclease. H2A.W is found in heterochromatin (Yelagandula et al., 2014), where it may serve a silencing function. Like H2A.X, it can be phosphorylated during the DNA damage response (Lorković et al., 2017). Extended C-terminal tails are also found in H2As of bdelloid rotifers, freshwater microorganisms, which replace conventional H2A, H2A.X and H2A.Z, and are speculated to help protect against DNA damage from desiccation (Van Doninck et al., 2009). In animals, the macroH2A variant acts like H2A.W in protecting 10 bp of extranucleosomal DNA and is distinguished from other H2As in that the HFD is connected to a separate macrodomain by a basic protein linker region (Chakravarthy et al., 2012). Heterotypic macroH2A–H2A nucleosomes form a more stable octamer (Bowerman et al., 2019), and the linker region facilitates condensation (Muthurajan et al., 2011). MacroH2A nucleosomes have reduced recruitment of chromatin remodelers, inhibit acetylation by p300 (also known as EP300) (Chang et al., 2008; Doyen et al., 2006a) and present a barrier to reprogramming cells (Pliatska et al., 2018) by stabilizing both active and inactive gene expression patterns.

In contrast to H2A.W and macroH2A, four families of short H2As (H2A.B, H2A.L, H2A.P and H2A.Q), which wrap only 110–130 bp of DNA and have shortened docking domains, reduced DNA-binding capability and smaller acidic patches, are encoded on the X chromosome of placental mammals (Bao et al., 2004; Dai et al., 2018; Molaro et al., 2018). All these families have stage-specific expression in testes, where they have roles in splicing and the transition to protamines, and are evolving rapidly (Table 1).

## H1 variants

Linker histone H1 lacks a HFD and has a different origin than the other histones (Kasinsky et al., 2001). It is absent in the early-diverging metamonads (Dalmasso et al., 2011) and it is unclear whether it was present in the last eukaryotic common ancestor. In multicellular eukaryotes, H1s have a tripartite structure in which a globular domain with a winged helix motif separates basic N-terminal and C-terminal domains that are variable. The lysine-rich C-terminus, which often contains S/TPKK minor groove-binding motifs, is similar to lysine-rich DNA-binding proteins in bacteria and is necessary for chromatin compaction (Healton et al., 2020; Kasinsky et al., 2001). The winged helix, which binds at the nucleosome dyad and interacts with the entry and exit linker DNAs (Bednar et al., 2017) (see poster), was either independently acquired in plants, mycetozoans and opisthokonts (animals, fungi and near relatives), or independently lost in kinetoplastids, alveolates and *Entamoeba* (Kasinsky et al., 2001).

Humans and other mammals have 11 H1 paralogs (seven somatic paralogs and four germline paralogs). The genes encoding the somatic paralogs H1.1–H1.5 and the ‘testis-specific’ variant H1.6 (H1t) are part of the histone gene cluster on chromosome VI, whereas RI variants H1.0 and H1.7–H1.10 (H1T2, H1oo, HILS1 and H1X) are encoded elsewhere. In ChIP-seq experiments of endogenous or HA-tagged H1.0, H1.2–H1.5 and H1.10 in a breast cancer cell line, H1s are broadly found on genes, repeats and upstream promoters, but are depleted at transcription start sites (Millán-Ariño et al., 2014). In human lung fibroblasts, H1.5 is enriched over splice sites of exons shorter than the length of a nucleosome and promotes their inclusion, apparently by stalling RNAPII (Glaich et al., 2019). In mouse embryonic stem cells, H1.2 and H1.3 (H1c and H1d) are enriched in heterochromatic domains marked with H3K9me3, and *in vitro* H1.0–H1.5 interact directly with H3K9 methyltransferases through their C-termini and promote H3K9 methylation (Healton et al., 2020) (see poster). Knockout of individual H1s generally has little effect on mouse development, but triple knockout of H1.2, H1.3 and H1.4 (H1e) results in embryonic lethality (Fan et al., 2003). In an embryonic cell line derived from the triple knockout, with only 20% of global H1 expression, strong de-repression of transcription of the major satellite and other repeat classes occurs together with loss of H3K9me3 on the affected sequences (Healton et al., 2020). Although H1 variants have at least partially redundant functions, they show different effects on nucleosome spacing when introduced to H1-free *Xenopus* oocytes, with H1.2 and H1.3 increasing nucleosome repeat length by only 5–7 bp, whereas H1.4 and H1.0 from *Xenopus* and chicken (H5) increase nucleosome repeat length by 13–20 bp (Öberg et al., 2012). Short repeat length results in greater compaction and silencing (Healton et al., 2020).

The somatic RI variants H1.0 and H1.10 are enriched at nucleolus-associated domains and at RNAPII-enriched domains, respectively (Mayor et al., 2015). H1.0 is conserved in vertebrates and invertebrates, and is found primarily in differentiated tissues (González-Romero et al., 2009). H1.0-binding sites positively correlate with the presence of H3K27me3 (a mediator of developmental silencing), high nucleosome density and GC-rich genes in fibroblasts, and are at low density in AT-rich regions (Torres et al., 2016). H1.0 is often heterogeneously expressed in tumor cells, with H1.0 levels correlating with tumor cell differentiation and patient survival, whereas silencing of H1.0 favors self-renewing cells. Similarly, lower levels of H1.10 are an adverse prognosticator for astrocytic gliomas (Sepsa et al., 2015).

## Perspective

From an ancestral set of five proteins, four of which are among the most conserved proteins known, histone variants continue to diversify and innovate to respond to the necessity of regulating access to DNA in all the contexts in which organisms find themselves. Histone variants greatly expand the roles and dynamics of nucleosomes by wrapping more or less DNA, by having greater or lesser stability, by having unique post-translational modifications or by interacting with other chromatin components. Although ancient variants, such as H2A.Z and H3.3, are well-studied, they continue to raise questions – are their effects on transcription and silencing cellular adaptations to their chromatin maintenance functions in non-dividing cells, when RC histones are unavailable? Similar questions arise in considering the DNA repair-independent functions of H2A.X. More recently evolved variants raise additional questions, such as what is the role of the enigmatic macroH2A domain, the only globular domain fused to a histone, and which is unique to animals. Especially intriguing are the sperm-specific variants in every histone class, which are collectively involved in the process of packaging the genome into protamines, but their individual roles are only starting to become clear. Other challenges include understanding how dysregulation or mutation of histone variants and their chaperones promote tumorigenesis (Bennett et al., 2019; Lowe et al., 2019; Nacev et al., 2019; Skene and Henikoff, 2013). New profiling and gene editing techniques promise to address these challenges.
